# The social value of a PASI 90 or PASI 100 response in patients with moderate-to-severe plaque psoriasis in Spain

**DOI:** 10.3389/fpubh.2023.1000776

**Published:** 2023-01-26

**Authors:** Paulina Maravilla-Herrera, María Merino, Santiago Alfonso Zamora, Jesús Balea Filgueiras, José Manuel Carrascosa Carrillo, Olga Delgado Sánchez, Francisco Dolz Sinisterra, Antonio García-Ruiz, Pedro Herranz Pinto, Antonio Manfredi, José Martínez Olmos, Paloma Morales de los Ríos Luna, Lluís Puig, Sandra Ros, Álvaro Hidalgo-Vega

**Affiliations:** ^1^Department of Health Outcomes Research, Weber, Madrid, Spain; ^2^Department of Management, Psoriasis and Psoriatic Arthritis Patient and Family Association (Acción Psoriasis), Barcelona, Spain; ^3^Department of Pharmacy, Ferrol University Hospital Complex, La Coruña, Spain; ^4^Department of Dermatology, Germans Trias i Pujol University Hospital, Badalona, Spain; ^5^Department of Management, Spanish Society of Hospital Pharmacy (SEFH), Madrid, Spain; ^6^Department of Pharmacy, Son Espases University Hospital, Illes Balears, Spain; ^7^Department of Management, Doctor Peset University Hospital, Valencia, Spain; ^8^Health Economics and Rational Use of Medicines, Department of Pharmacology and Clinical Therapeutics, Biomedical Research Institute of Malaga (IBIMA), University of Malaga, Malaga, Spain; ^9^Department of Dermatology, La Paz University Hospital, Madrid, Spain; ^10^Andalusian Public Health School (EASP), Granada, Spain; ^11^Department of Nursing, Gregorio Marañón University Hospital, Madrid, Spain; ^12^Department of Dermatology, Santa Creu i Sant Pau Hospital, Barcelona, Spain; ^13^Universitat Autònoma de Barcelona, Barcelona, Spain; ^14^Psychologist, Departments of Dermatology and Rheumatology, and Cardiac Transplant Unit, Santa Creu i Sant Pau Hospital, Barcelona, Spain; ^15^Department of Economic Analysis and Finances, University of Castilla-La Mancha, Toledo, Spain; ^16^Fundación Weber, Madrid, Spain

**Keywords:** social return, socioeconomic impact, psoriasis, Psoriasis Area and Severity Index (PASI), quality of life, out-of-pocket (OOP) expenses, activities of daily living

## Abstract

**Introduction:**

Psoriasis is a chronic disease involving the skin, which significantly impacts the quality of life. Disease severity and treatment efficacy (i.e., response) are assessed through the Psoriasis Area and Severity Index (PASI). A PASI 75 response, i.e., an improvement of at least 75% with respect to the baseline PASI score, has traditionally been used as a therapeutic benchmark in clinical trials. Therapeutic advances have made PASI 90 or PASI 100 responses possible in most patients treated with some biologics. A greater response may generate social value beyond clinical outcomes that would benefit both patients and society.

**Methods:**

A 1-year economic model was applied to estimate the impact of having a PASI 75, PASI 90, or PASI 100 response in four areas of analysis (quality of life, activities of daily living, work productivity, and out-of-pocket expenditures) and the social value of having a PASI 90 or PASI 100 response in comparison with a PASI 75 response. A mixed-methods approach based on the scientific literature, a focus group with patient, and an advisory committee with psoriasis stakeholders was used. The model included three different scenarios: having a PASI 90 vs a PASI 75 response; a PASI 100 vs a PASI 90 response; and a PASI 100 vs a PASI 75 response. A sensitivity analysis was included.

**Results:**

The annual economic impact per patient with moderate-to-severe plaque psoriasis having a PASI 75 response was estimated at Ł 6,139, mainly related to labour productivity losses and quality of life reductions. Having a PASI 90 or a PASI 100 response would reduce this impact to €3,956 or €1,353, respectively. Accordingly, the social value of having a PASI 90 instead of a PASI 75 response was estimated at €2,183, and €4,786 with a PASI 100 response.

**Discussion:**

A PASI 90 or PASI 100 response would have a lower economic impact and a greater social value than a PASI 75 response for patients with moderate-to-severe plaque psoriasis.

## 1. Introduction

Psoriasis is a chronic non-communicable disease that can cause systemic inflammation and is associated with multiple comorbidities (1). It is a painful, pruritic, and disabling disease that involves the skin and nails, causing a significant impact on the quality of life of patients (1). Psoriasis can occur at any age but normally appears before the age of 40 (2). Approximately 2.3% of the Spanish population is affected by this condition; plaque psoriasis is the most common form of psoriasis accounting for 90% of all cases (3, 4).

The clinical diagnosis of psoriasis is based on the assessment of the patient's history and the presence of skin lesions with characteristic morphology and distribution (5). Disease severity is usually assessed through the Psoriasis Area and Severity Index (PASI), which combines an evaluation of the severity of the lesions with erythema, infiltration, and peeling, scored on a scale of 0 to 4 (0 = none, 1 = mild, 2 = moderate, 3 = marked, and 4 = very marked) and the percentage of skin involved on the head, trunk, upper limbs, and lower limbs. Moderate or severe disease can be defined by a PASI score of 5–10 or a PASI score of >10, respectively (6). In Spain, 29.5% of patients with psoriasis have a diagnosis of moderate-to-severe plaque psoriasis (7). According to this data, nearly 300,000 inhabitants of Spain suffer from moderate-to-severe plaque psoriasis (7).

Moreover, the PASI is also used to assess the efficacy of treatment. For example, a PASI 75 response means an improvement in the PASI score of at least 75% with respect to the baseline (pre-treatment) PASI score (6). Previously, achieving a PASI 75 response was considered a relevant therapeutic goal, yet nowadays achieving a PASI 90 response or even a PASI 100 response is possible given recent therapeutic advances (5, 8–10). Accordingly, the optimal therapeutic response may be defined by the equivalent of PASI 90 or PASI 100 responses in clinical trials. The course of the disease, the morphology, the severity, and the body surface area involved are different for each patient and may not only have an impact on the clinical dimension but also the personal, work-life, social, and intimacy dimensions of the patient (10–13). Therefore, disease management should be personalized and patient-centered, with a clear therapeutic goal agreed upon between clinician and patient (1).

According to the literature, benefits outside the clinical dimension may be achieved by setting more ambitious therapeutic objectives, as patients with improved skin clearance further report emotional, psychological, and social functioning improvements (14–16). Accordingly, the economic impact (i.e., related costs) associated with a poor quality of life or work productivity losses in psoriasis patients who have not achieved an optimal therapeutic response may be reduced by increasing the degree of response. Therefore, achieving a PASI 90 or PASI 100 response in patients with moderate-to-severe plaque psoriasis may reduce this economic impact, which translates into a social value gain. Estimating the social value from a holistic perspective can help to understand how achieving a greater PASI response may improve different aspects of the patients' lives, considering both tangible and intangible aspects that are meaningful for patients.

To our knowledge, no other study to date has analyzed the social value of achieving a PASI 90 or PASI 100 response in patients with moderate-to-severe plaque psoriasis in Spain.

## 2. Materials and methods

**Figure 1:**
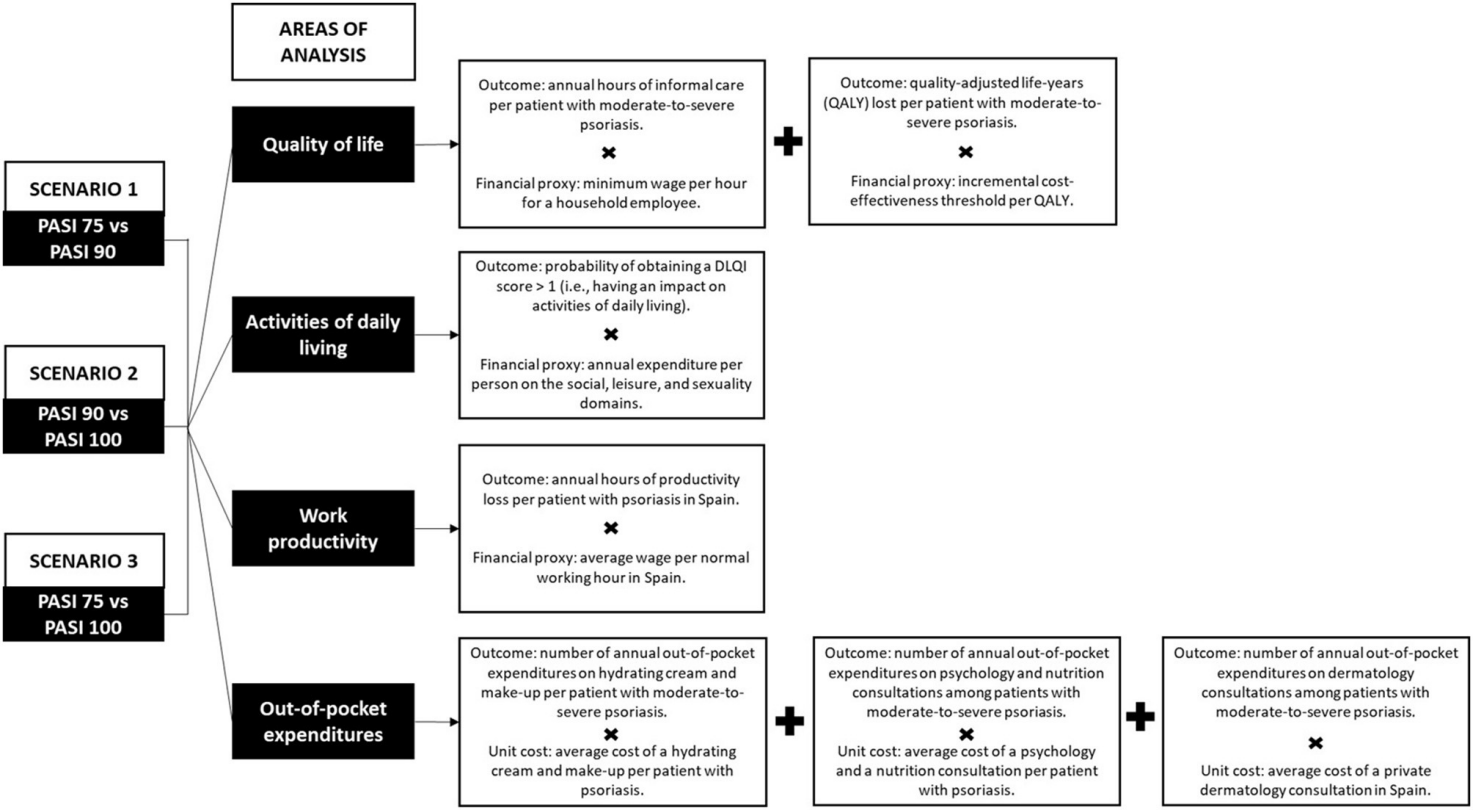
PASI scenarios and areas of analysis.

An economic model with a one-year time horizon was applied to estimate the economic impact of having achieved a PASI response of 75, 90 or 100, and the social value of having achieved a complete or almost complete response in patients with moderate-to-severe plaque psoriasis in Spain. The economic impact was estimated from a social perspective and included costs related to the following four areas of analysis: (1) reduction in quality of life (including the need for informal care and quality-adjusted life-years lost because of the disease); (2) impact on activities of daily living, defined by the activities patients stop doing or their willingness to pay to improve this sphere; (3) work productivity losses, i.e., missing working hours due to the disease (medical visits and/or sick leaves); and (4) psoriasis-related out-of-pocket expenditures; in three different response scenarios: (1) a PASI 75 response compared to a PASI 90 response; (2) a PASI 90 response compared to a PASI 100 response; and (3) a PASI 75 compared to a PASI 100 response (Figure 1).

The economic impact generated by a patient with a PASI 75, PASI 90, or PASI 100 response was estimated by multiplying the outcomes associated with presenting each response (Table 1) by their financial value or financial proxy (Table 2) within each area of analysis (Figure 1). Moreover, the social value was estimated by subtracting the economic impact (i.e., associated costs) of presenting a PASI 75 or PASI 90 response from the economic impact of a more complete response, according to the three different scenarios (Figure 1). Both the economic impact and the social value are reported per patient and year, expressed in 2021 euros (€). Unit prices prior to 2021 were updated to the year 2021 according to the corresponding general or medical Consumer Price Index whenever needed (20).

**Table 1:** Outcomes by areas of analysis and PASI responses.

**Outcome**	**PASI 75**	**PASI 90**	**PASI 100**
**Quality of life**			
*(a) Probability of obtaining a PSI score > 0 (i.e., presenting severe signs and symptoms) (14).^‡^*	*0.86*	*0.74*	*0.35*
*(b) Average days per year in which patients with psoriasis on biological treatment needed assistance (17)*.	*9.60*	*9.60*	*9.60*
*(c) Hours of informal care per day in which a patient with moderate-to-severe psoriasis needs assistance.^†^*	*4.00*	*2.00*	*0.00*
**Annual hours of informal care per patient with moderate-to-severe psoriasis. (a** ^*^ **b** ^*^ **c)**	**33.10**	**14.15**	**0.00**
*(a) Average utility of the Spanish population (18).^#^*	*0.912*	*0.912*	*0.912*
*(b) Average utility of patients with moderate-to-severe psoriasis (19)*.	*0.891*	*0.900*	*0.902*
**Quality-adjusted life-years lost per patient with moderate-to-severe psoriasis. (a-b)**	**0.021**	**0.012**	**0.010**
**Activities of daily living**			
**Probability of obtaining a DLQI score** **>** **1 (i.e., having an impact on activities of daily living)** **(****20****)**.^‡^	**0.48**	**0.26**	**0.13**
**Work productivity**			
*(a) Probability of having a job among patients with moderate-to-severe psoriasis (21)*.	*0.65*	*0.65*	*0.65*
(b) Annual hours of productivity loss per working patient with psoriasis in Spain.^‡^ (i/ii^*^iii)	*474.33*	*315.31*	*87.00^†^*
*(i) Annual hours of productivity loss per patient with psoriasis in the UK (22)*.	*179.92*	*119.60*	*NA*
*(ii) Annual hours of productivity loss per worker in the UK (23).^#^*	*33.00*	*33.00*	*NA*
*(iii) Annual hours of productivity loss per worker in Spain (24).^#^*	87.00	*87.00*	*87.00*
**Annual hours of productivity loss per patient with psoriasis in Spain. (a** ^*^ **b)**	**308.32**	**204.95**	**56.55**
**Out-of-pocket expenditures**			
*(a) Probability of having out-of-pocket expenditures on hydrating cream and make-up among patients with moderate-to-severe psoriasis (25)*.	*0.49*	*0.44* ^†^ ^*^	*0.40* ^†^ ^*^
*(b) Number of annual out-of-pocket expenditures on hydrating cream and make-up per patient with moderate-to-severe psoriasis requiring them.^†^^‡^*	*4.00*	*4.00*	*4.00*
**Number of annual out-of-pocket expenditures on hydrating cream and make-up per patient with moderate-to-severe psoriasis. (a** ^*^ **b)**	**1.97**	**1.78**	**1.58**
*(a) Probability of having out-of-pocket expenditures on psychology and nutrition among patients with moderate-to-severe psoriasis (25)*.	*0.11*	*0.99* ^†^ ^*^	*0.88* ^†^ ^*^
*(b) Number of annual out-of-pocket expenditures on psychology and nutrition consultations among patients with moderate-to-severe psoriasis requiring them.^†^^‡^*	*4.00*	*4.00*	*4.00*
**Number of annual out-of-pocket expenditures on psychology and nutrition consultations among patients with moderate-to-severe psoriasis. (a** ^*^ **b)**	**0.44**	**0.39**	**0.35**
*(a) Probability of having out-of-pocket expenditures on dermatology consultations among patients with moderate-to-severe psoriasis (25)*.	*0.036*	*0.032* ^†^ ^*^	*0.029* ^†^ ^*^
*(b) Number of annual out-of-pocket expenditures on dermatology consultations among patients with moderate-to-severe psoriasis requiring them.^†^^‡^*	*2.00*	*2.00*	*2.00*
**Number of annual out-of-pocket expenditures on dermatology consultations among patients with moderate-to-severe psoriasis. (a** ^*^ **b)**	**0.072**	**0.065**	**0.058**

† Assumption.

‡ Included in the sensitivity analysis.

# PASI was not considered. NA: not applicable, as the annual hours of productivity loss per worker in Spain were assumed for patients with a PASI 100 response.

* The probability used in PASI 90 and PASI 100 was reduced by 10 and 20% with respect to PASI 75, respectively. DLQI, Dermatology Life Quality Index; NA, not applicable; PSI, Psoriasis Symptom Inventory (a patient-reported outcome measure for assessing the severity of plaque psoriasis symptoms). Bold values represent the final equation, and italic values (exemplified by letters) represent the variables taken into account in the final equation.

**Table 2:** Financial values or proxies by areas of analysis.

**Financial value or financial proxy**	**Unit cost**
**Quality of life**	
**Minimum wage per hour for a household employee, as a financial proxy for an hour of informal care** **(****26****)**.^†^	€ **7.43**
**Incremental cost-effectiveness threshold per quality-adjusted life-year, as a financial proxy for quality of life** **(****27****)**.^‡^	€ **21,000.00**
**Activities of daily living**	
*(a) Average annual expenditure per person on bars and cafeterias in Spain (28).^†^*	€* 260.00*
*(b) Average annual expenditure per person on recreational, sports, and cultural activities (28).^†^*	€* 117.42*
*(c) Willingness to pay per patient with moderate-to-severe psoriasis for an improvement in their sexual life (21).^†^*	€* 745.80*
**Annual expenditure per person on the social, leisure, and sexuality domains. (a** **+** **b** **+** **c)**	€ **1,123.22**
**Work productivity**	
**Average wage per normal working hour in Spain** **(****29****)**.	€ **15.43**
**Out-of-pocket expenditures**	
(a) Average cost of a hydrating cream for psoriasis.^*^	€* 10.00*
(b) Average cost of make-up to cover up psoriasis lesions.^*^	€* 45.00*
**Average cost of a hydrating cream and make-up per patient with psoriasis. (a+b)** *^**#**^*	€ **55.00**
(a) Average cost of a private psychology consultation in Spain.^*^	€* 50.00*
(b) Average cost of a private nutrition consultation in Spain.^*^	€* 50.00*
**Average cost of a psychology and a nutrition consultation per patient with psoriasis. (a+b)** *^**#**^*	€ **100.00**
**Average cost of a private dermatology consultation in Spain**. ^*^	€ **100.00**

† Financial proxy.

‡ Included in the sensitivity analysis.

# Reference cost for PASI 75 response. A lower amount has been estimated for patients with PASI 90 and PASI 100 response (10% and 20% respectively).

* Market price. QALY, quality-adjusted life-year. Bold values represent the final equation, and italic values (exemplified by letters) represent the variables taken into account in the final equation.

The economic model was developed using a mixed-methods approach to gather relevant information from the scientific literature, a focus group (FG) with patients, and an advisory committee with experts. First of all, a literature review was carried out on the PubMed^®^ search engine by combining PASI response terms (PASI 75, PASI 90, and PASI 100) and their synonyms with an extensive list of impact terms (cost, resource, impairment, pain, itch, burn, sting, cracking, redness, scaling, flaking, anxiety, depression, caregiving, work productivity, activities of daily living, quality of life, suicide, bullying, sex, relationships, and leisure) and their synonyms through AND/OR Boolean operators, to identify and extract data on the economic impact of having achieved a PASI 75, PASI 90, or PASI 100 response. The search retrieved 383 studies of which 14 were selected as they included relevant information in line with the purpose of the study. These studies did not include information on the impact on healthcare resource consumption and costs generated by patients with different PASI responses.

Thereafter, a FG comprising six patients with moderate-to-severe psoriasis from different regions in Spain was carried out to understand the impact of the disease on the patients' lives, especially within the four areas of analysis, their unmet needs, their willingness to pay for symptom improvement, and their perceived benefits of having obtained a PASI 90 or PASI 100 response in terms of quality of life, physical and emotional state, labor productivity, activities of daily living, informal care, and out-of-pocket expenditures. Finally, an advisory committee comprising a multidisciplinary group of twelve experts representing the main stakeholders regarding psoriasis in Spain (dermatologists, nurses, psychologists, pharmacists, hospital managers, health economists, policymakers, and patients) was created to discuss and agree on the most appropriate inputs, obtained from the literature review and the FG (e.g., study selection based on the quality of the methods), that would be included in the model to quantify the social value of a PASI 90 or PASI 100 response with respect to a PASI 75 response.

In the absence of relevant data, sensible assumptions were made following a conservative approach, supported by the scientific literature and validated by the advisory committee. A sensitivity analysis was carried out to test the strength of the model when varying assumptions as follows: the reference scenario, the lower limit or best-case scenario (that which would result in a lower impact) and the upper limit or worst-case scenario (that which would result in a greater impact). Outcomes, financial values, or financial proxies included in the sensitivity analysis are displayed in Table 3.

**Table 3:** Outcomes, financial values, or financial proxies included in the sensitivity analysis.

**Outcome, financial value, or financial proxy**	**Worst-case scenario**	**Reference scenario**	**Best-case scenario**
**Quality of life**			
Probability of obtaining a PSI score > 0 with a PASI 75 response (14).	0.78^†^	0.86	0.95^‡^
Probability of obtaining a PSI score > 0 with a PASI 90 response (14).	0.66^†^	0.74	0.81^‡^
Probability of obtaining a PSI score > 0 with a PASI 100 response (14).	0.32^†^	0.35	0.39^‡^
Incremental cost-effectiveness threshold per quality-adjusted life-year (27).	€ 11,000	€ 21,000	€ 30,000
**Activities of daily living**			
Probability of obtaining a DLQI score > 1 with a PASI 75 response (20).	0.43^†^	0.48	0.52^‡^
Probability of obtaining a DLQI score > 1 with a PASI 90 response (20).	0.23^†^	0.26	0.29^‡^
Probability of obtaining a DLQI score > 1 with a PASI 100 response (20).	0.11^†^	0.13	0.14^‡^
**Work productivity**			
Annual hours of productivity loss per patient with psoriasis in Spain with a PASI 75 response (22–24).	426.90	474.33	521.77
Annual hours of productivity loss per patient with psoriasis in Spain with a PASI 90 response (22–24).	283.78	315.31	346.84
Annual hours of productivity loss per patient with psoriasis in Spain with a PASI 100 response (24).	78.30	87.00	95.70
**Out-of-pocket expenditures**			
Number of annual out-of-pocket expenditures on hydrating cream or make-up per patient with moderate-to-severe psoriasis requiring them.^#^	2.00	4.00	6.00
Number of annual out-of-pocket expenditures on psychology or nutrition consultations among patients with moderate-to-severe psoriasis requiring them.^#^	2.00	4.00	6.00
Number of annual out-of-pocket expenditures on dermatology consultations among patients with moderate-to-severe psoriasis requiring them.^#^	1.00	2.00	3.00

† Variation of−10% with respect to the reference scenario.

‡ Variation of +10% with respect to the reference scenario.

# Assumption. Abbreviations: DLQI, Dermatology Life Quality Index; PASI, Psoriasis Area Severity Index; PSI, Psoriasis Symptom Inventory (a patient-reported outcome measure for assessing the severity of plaque psoriasis symptoms).

Given the nature of this study, approval by an institutional review board or an ethical review board was not required. Nevertheless, study procedures were in accordance with the Declaration of Helsinki 1975/83.

## 3. Results

The annual economic impact of having a PASI 75 response in patients with moderate-to-severe psoriasis was estimated at € 6,139 per patient. A PASI 90 response would result in a smaller economic impact (€ 3,956) and even lower with a PASI 100 response or completely clear skin, estimated at € 1,353 per patient (Table 4).

**Table 4:** Economic impact by area of analysis and PASI response in the reference scenario.

	**PASI 75**	**PASI 90**	**PASI 100**
**Annual cost of the impact on quality of life (a** **+** **b)**	€ 686.94	€ 357.14	€ 210.00
*(a) Quality of life: costs associated with the need for informal care*	€* 245.94*	€* 105.14*	€* 0.00*
*(b) Quality of life: costs associated with quality-adjusted life-years lost*	€* 441.00*	€* 252.00*	€* 210.00*
Annual cost of the impact on activities of daily living	€ 533.53	€ 292.04	€ 142.65
Annual cost of work productivity loss	€ 4,758.81	€ 3,163.37	€ 872.84
Annual cost of out-of-pocket expenditures	€ 159.61	€ 143.64	€ 127.68
Total annual cost per patient	€ 6,138.88	€ 3,956.19	€ 1,353.17

Bold values represent the final equation, and italic values (exemplified by letters) represent the variables taken into account in the final equation.

Regarding the quality of life associated with the need for informal care, a PASI 75 response would involve an annual cost of € 246, higher than the estimated cost associated with a PASI 90 response (€ 105). On the contrary, patients with PASI 100 response would not generate informal care costs. Similarly, the economic impact associated with the number of quality-adjusted life-years lost by patients with moderate-to-severe psoriasis would be higher in those with a PASI 75 response (€ 441) than in those with a PASI 90 or PASI 100 response (€ 252 and € 210, respectively) (Table 4).

Concerning the impact of moderate-to-severe psoriasis in activities of daily living, according to the results in Table 4, a PASI 75 response would have a higher economic impact (€ 534) than a PASI 90 (€ 292) or PASI 100 response (€ 143).

Regarding work productivity losses in patients with moderate-to-severe psoriasis, the associated economic impact would be higher in those patients with a PASI 75 response (€ 4,759), compared with a PASI 90 (€ 3,163) and PASI 100 response (€ 873) (Table 4).

Finally, patients with a PASI 75 response would generate an annual cost of € 160 in out-of-pocket expenditures, slightly more than those with a PASI 90 or PASI 100 response (€ 144 and € 128, respectively) (Table 4).

**Figure 2:**
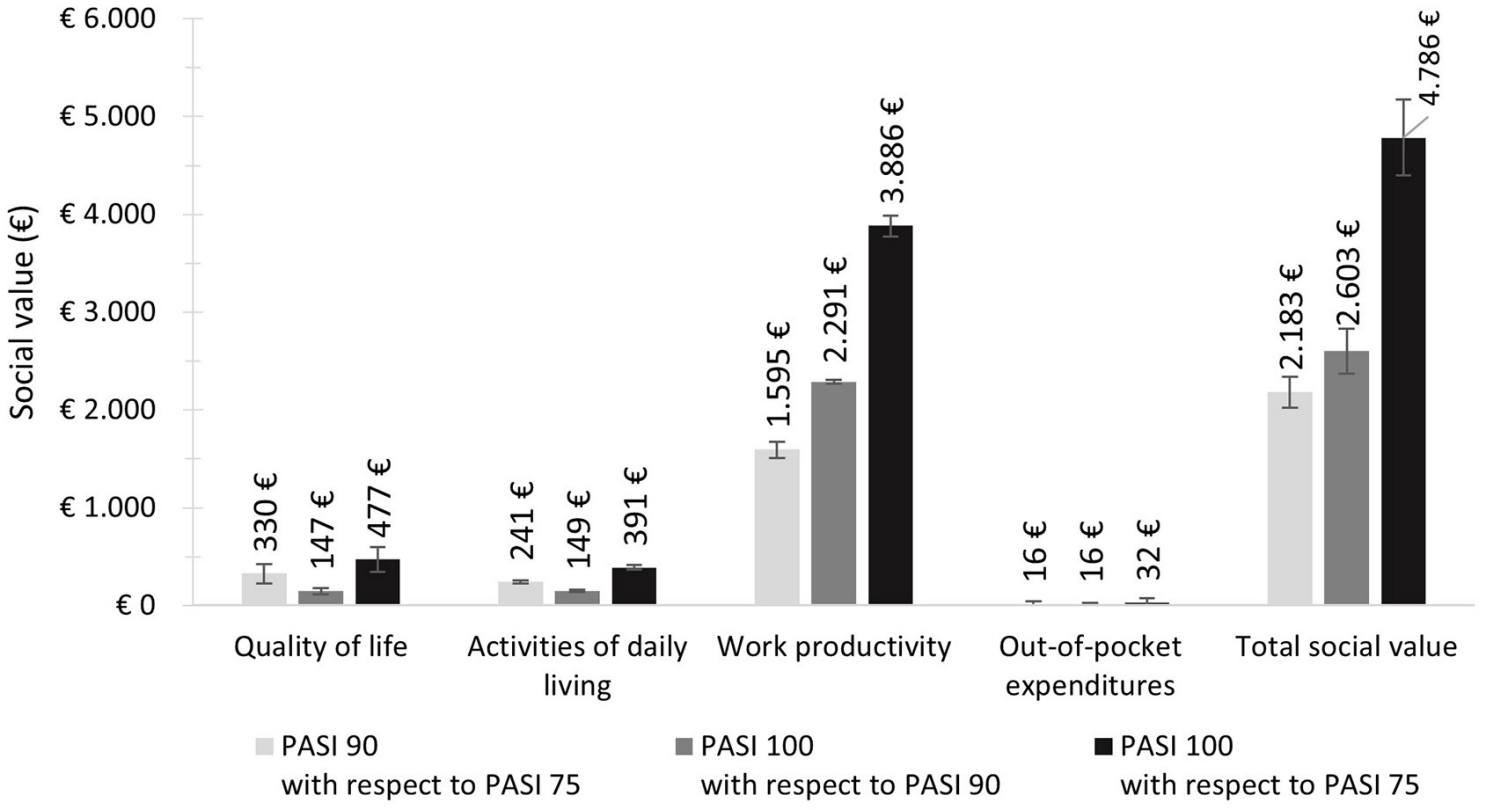
Potential social value according to PASI scenarios, areas of analysis, and total.

Concerning the social value, obtaining a PASI 90 response instead of a PASI 75 response would represent an added social value of € 2,183 and more than double with a PASI 100 response (€ 4,786). Additionally, obtaining a PASI 100 response instead of a PASI 90 response would imply an additional social value of € 2,603 (Figure 2). This tendency was observed in all four areas of analysis.

**Figure 3:**
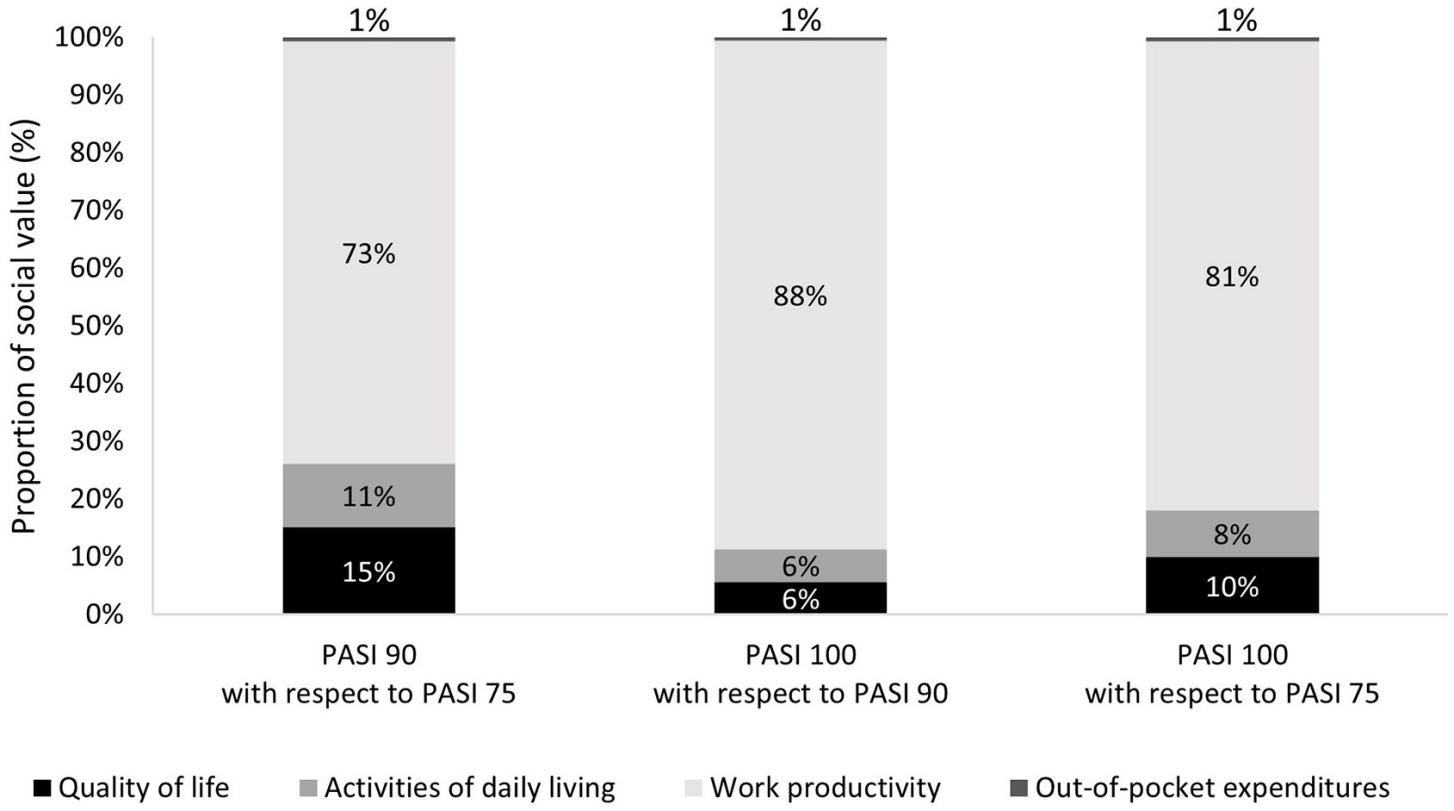
Distribution of social value according to areas of analysis and PASI scenarios.

The greatest proportion of the social value attained when obtaining a PASI 90 or PASI 100 response instead of a PASI 75 response would be attributed to work productivity gains (73.1 and 81.2% of the social impact, respectively). Similarly, 88% of the social value attained when obtaining a PASI 100 response instead of a PASI 90 response would be attributed to work productivity gains. Improvements in the quality of life, activities of daily living, and reductions in out-of-pocket expenditures would account for 15.1, 11.1, and 0.7% of the total social value attained when obtaining a PASI 90 response instead of a PASI 75 response, and for 10, 8.2, and 0.7% of the social value attained when obtaining a PASI 100 response instead of a PASI 75 response. For those patients obtaining a PASI 100 response instead of a PASI 90 response, the social value in these areas would be 5.7, 5.7, and 0.6%, respectively (Figure 3).

According to the sensitivity analysis, the greatest variability of the economic impact was associated with a PASI 100 response, which could decrease by 20% to € 1,088, or increase by 23% to € 1,609. Meanwhile, the economic impact associated with a PASI 75 or PASI 90 response could decrease by 14% in the worst-case scenario or increase by 16% in the best-case scenario. The corresponding social value would vary accordingly.

## 4. Discussion

Having a PASI 90 or PASI 100 response would generate a higher social value and have a lower economic impact for patients with moderate-to-severe plaque psoriasis compared with a PASI 75 response. According to the overall results, the economic impact of a PASI 75 response would be 66 and 78% higher than PASI 90 or 100 response, respectively.

In terms of quality of life, this study aligns with previous research indicating that psoriasis patients with greater improvements from the baseline PASI scores are less severely affected, have reduced symptomatology, and consequently present a better quality of life (14, 20, 30). On one hand, this impact may be reflected as the cost of informal care or assistance in activities of daily living. According to the information collected from the FG and the scientific literature, psoriasis symptomatology interferes with household and basic activities, therefore some patients may need help to accomplish them (17). On the other hand, Pickard et al. (19) indicated that the utility of patients that achieved a PASI 75 response was slightly smaller than for patients who achieved a PASI 90 or PASI 100 response. Regarding the quality of life, the results of the present study estimated that if a patient achieves a PASI 90 or 100 response, instead of a PASI 75 response, € 330 and € 477 of social value would be generated per year, respectively.

According to the patients in the FG, the area of activities of daily living generates the largest impact on patients' lives. Not being able to do activities because of lesions in exposed areas (e.g., sports or having intimate relationships, among others) may have a great psychological impact or lead to stigmatization (31). Contrary to the information collected in the FG, this area was not one of major impact, possibly associated with its subjectiveness and difficulty of assessment. Accordingly, results may not reflect the whole impact this area represents on patients. One of the most frequently used methods to assess this is contingent valuation through the willingness to pay. Several studies have observed that in patients with dermatologic conditions, willingness to pay for improving certain spheres of their lives (including activities of daily living) could vary when treatment improved their condition (32–35). Accordingly, the results of the present study showed a greater economic impact associated with a PASI 75 response (€ 534) than that of a PASI 90 (€ 292) or PASI 100 response (€ 143).

The greatest impact of psoriasis has been associated with the area of work productivity, as previous studies have shown that productivity losses reflect the overall impact of psoriasis (22, 36, 37). According to our results, achieving a PASI 90 or PASI 100 response, compared to a PASI 75 response, would translate into an additional social value of € 1,595 and € 3,886, respectively.

Concerning out-of-pocket expenditures, despite psoriasis treatment being covered by the Spanish National Health System, there is still room for improvement. Health services may vary depending on the area of residence or may not cover all of the patient's needs, having them pay to address them on their own (38). According to Richard et al. (25) out-of-pocket expenditures of patients with psoriasis in France were more than twice the expenditures for other medical conditions. In Spain, patients in the FG mentioned that their out-of-pocket expenditures are usually related to private health care (dermatology, psychology, and nutrition), personal care products or services (hydrating cream, make-up, and hairstyling), or special clothing, among others. This situation may imply private financing from the patients that, according to the results, may have a greater impact on patients who only achieve PASI 75 response.

The present study has some limitations. First, there was a lack of scientific literature regarding the impact of psoriasis on patients' lives according to different levels of the PASI response. Relevant information was found in only 14 studies out of 383 revised. Specifically, there was a lack of information on healthcare resource consumption and costs, work productivity, and out-of-pocket expenditures in Spanish psoriasis patients: regarding work productivity losses, British figures were extrapolated to the Spanish context; concerning out-of-pocket expenditures, only those mentioned by patients in the FG were considered. Moreover, the reduced number of studies and the large differences between them further hindered the use of statistical analyses to compare PASI responses. Second, the impact of psoriasis may vary from patient to patient and hence be experienced in different ways according to each reality. Therefore, a conservative approach was adopted when quantifying the economic impact of the disease and the social value of achieving better PASI responses. Finally, despite the small number of representatives of psoriasis patients in the FG and experts in the advisory committee, all data and information collected from these groups were supported by scientific literature. The results in this study should be taken with precaution when interpreted, always considering the sensitivity analysis. In this regard, the sensitivity analysis considered variations in most especially relevant parameters (i.e., ±10% for probabilities), variations based on the scientific literature available, and variations for assumptions based on information provided by the FG. Including the remaining variables in the sensitivity analysis to test the strength of the model would have implied making additional assumptions that may have biased the results and hindered the conservative approach of this study.

Despite these limitations, this study adds an understanding of the impact of the disease from the patient's perspective. Several studies have highlighted the importance of considering the social perspective in health economic analyses since it ideally captures health impacts that are particularly important for the understanding of health interventions and the effects on the population (39–41). Nevertheless, future studies are encouraged to assess the impact of new therapeutic goals on healthcare resource consumption and costs, with respect to the investment required to achieve such goals.

Considering the patient's view may add relevant information to psoriasis-related economic evaluations, giving a wide view of the impact of the disease from a multidimensional perspective.

## 5. Conclusions

Achieving PASI 90 or PASI 100 responses would have a lower economic impact, in comparison to a PASI 75 response, and hence generate a greater social value in terms of improved quality of life and activities of daily living, and reduced labor productivity losses and out-of-pocket expenditures for patients. This could be very significant not only for patients but also for their families and society at large.

## Data availability statement

The raw data supporting the conclusions of this article will be made available by the authors, without undue reservation.

## Author contributions

PM-H, MM, and ÁH-V conceived and designed the research. PM-H and MM acquired the data. PM-H, MM, SA, JB, JC, OD, FD, AG-R, PH, AM, JM, PM, LP, SR, and ÁH-V analyzed and interpreted the data. PM-H developed the original draft of the manuscript and MM substantively reviewed it. All authors critically revised and approved the manuscript.

## References

[1] World Health Organization. Global Report on Psoriasis. (2016). Available online at: https://apps.who.int/iris/handle/10665/204417 (accessed May 31, 2021).

[2] Langley RGB Krueger GG Griffiths CEM. Psoriasis: epidemiology, clinical features, and quality of life. Ann Rheum Dis. (2005) 64:ii18–23. 10.1136/ard.2004.033217 15708928 PMC1766861

[3] Griffiths CE Barker JN. Pathogenesis and clinical features of psoriasis. Lancet. (2007) 370:263–71. 10.1016/S0140-6736(07)61128-3 17658397

[4] Ferrándiz C Carrascosa JM Toro M. Prevalence of psoriasis in Spain in the age of biologics. Actas Dermo-Sifiliográficas. (2014) 105:504–9. 10.1016/j.adengl.2014.04.016 24569109

[5] Griffiths CEM Armstrong AW Gudjonsson JE Barker JNWN. Psoriasis. Lancet. (2021) 397:1301–15. 10.1016/S0140-6736(20)32549-6 33812489

[6] Grupo de Trabajo de Psoriasis de la Academia Española de Dermatología y Venereología (AEDV). ¿‘Cómo se mide la gravedad de la psoriasis? Principales índices.

[7] Carrascosa J Pujol R Daudén E Hernanz-Hermosa J Bordas X Smandia J. A prospective evaluation of the cost of psoriasis in Spain (EPIDERMA project: Phase II). J Eur Acad Dermatol Venereol. (2006) 20:840–5. 10.1111/j.1468-3083.2006.01659.x 16898908

[8] Strober BE van der Walt JM Armstrong AW Bourcier M Carvalho AV Chouela E. Clinical goals and barriers to effective psoriasis care. Dermatol Ther Heidelb. 9:5–18. 10.1007/s13555-018-0279-5 30578464 PMC6380974

[9] Armstrong AW Betts KA Signorovitch JE Sundaram M Li J Ganguli AX. Number needed to treat and costs per responder among biologic treatments for moderate-to-severe psoriasis: a network meta-analysis. Curr Med Res Opin. (2018) 34:1325–33. 10.1080/03007995.2018.1457516 29619856

[10] Ryan C Sadlier M De Vol E Patel M Lloyd AA Day A. Genital psoriasis is associated with significant impairment in quality of life and sexual functioning. J Am Acad Dermatol. (2015) 72:978–83. 10.1016/j.jaad.2015.02.1127 25824273

[11] Wahl A Loge JH Wiklund I Hanestad BR. The burden of psoriasis: a study concerning health-related quality of life among Norwegian adult patients with psoriasis compared with general population norms. J Am Acad Dermatol. (2000) 43:803–8. 10.1067/mjd.2000.107501 11050584

[12] Møller AH Erntoft S Vinding GR Jemec GB A. systematic literature review to compare quality of life in psoriasis with other chronic diseases using EQ-5D-derived utility values. Patient Relat Outcome Meas. (2015) 6:167–77. 10.2147/PROM.S81428 26185476 PMC4500621

[13] Villacorta R Teeple A Lee S Fakharzadeh S Lucas J McElligott S. multinational assessment of work-related productivity loss and indirect costs from a survey of patients with psoriasis. Br J Dermatol. (2020) 183:548–58. 10.1111/bjd.18798 31840228 PMC7497177

[14] Lacour J-P Bewley A Hammond E Hansen JB Horne L Paul C. Association between patient- and physician-reported outcomes in patients with moderate-to-severe plaque psoriasis treated with biologics in real life (PSO-BIO-REAL). Dermatol Ther. (2020) 10:1099–109. 10.1007/s13555-020-00428-1 32761560 PMC7477065

[15] Honma M Cai Z Burge R Zhu B Yotsukura S Torisu-Itakura H. Relationship between rapid skin clearance and quality of life benefit: post hoc analysis of japanese patients with moderate-to-severe psoriasis treated with Ixekizumab (UNCOVER-J). Dermatol Ther. (2020) 10:1397–404. 10.1007/s13555-020-00441-4 32910360 PMC7649171

[16] Puig L Thom H Mollon P Tian H Ramakrishna GS. Clear or almost clear skin improves the quality of life in patients with moderate-to-severe psoriasis: a systematic review and meta-analysis. J Eur Acad Dermatol Venereol. (2017) 31:213–20. 10.1111/jdv.14007 27739123

[17] Polistena B Calzavara-Pinton P Altomare G Berardesca E Girolomoni G Martini P. The impact of biologic therapy in chronic plaque psoriasis from a societal perspective: an analysis based on Italian actual clinical practice. J Eur Acad Dermatol Venereol JEADV. (2015) 29:2411–6. 10.1111/jdv.13307 26370321

[18] Instituto Nacional de Estadística. Encuesta Nacional de Salud 2011–2012. Cuestionario de Adultos. (2012). Available online at: https://www.mscbs.gob.es/estadisticas/microdatos.do (accessed Oct 30, 2018).

[19] Pickard AS Gooderham M Hartz S Nicolay C. EQ-5D health utilities: exploring ways to improve upon responsiveness in psoriasis. J Med Econ. (2017) 20:19–27. 10.1080/13696998.2016.1219359 27471948

[20] Blauvelt A Sofen H Papp K Gooderham M Tyring S Zhao Y. Tildrakizumab efficacy and impact on quality of life up to 52 weeks in patients with moderate-to-severe psoriasis: a pooled analysis of two randomized controlled trials. J Eur Acad Dermatol Venereol. (2019) 33:2305–12. 10.1111/jdv.15862 31407394 PMC6899626

[21] Carretero G Moreno D González Domínguez A Trigos D Ledesma A Sarquella E. Multidisciplinary approach to psoriasis in the Spanish National Health System: A social return on investment study. Glob Reg Health Technol Assess. (2020) 7:50–6. 10.33393/grhta.2020.2146 36627964 PMC9677591

[22] Warren RB Halliday A Graham CN Gilloteau I Miles L McBride D. Secukinumab significantly reduces psoriasis-related work impairment and indirect costs compared with ustekinumab and etanercept in the United Kingdom. J Eur Acad Dermatol Venereol JEADV. (2018) 32:2178–84. 10.1111/jdv.15094 29846965 PMC6586050

[23] Office for National Statistics. Sickness absence in the UK labour market—Office for National Statistics. (2020). Available online at: https://www.ons.gov.uk/employmentandlabourmarket/peopleinwork/labourproductivity/articles/sicknessabsenceinthelabourmarket/2020 (accessed Jan 2, 2022).

[24] Blasco de Luna FJ Barceló Larran D Blázquez Agudo EM Checa Martín JL Cirujano González A Pendás Pevida E. VIII Informe Adecco sobre absentismo. Madrid: The Adecco Group Institute. (2019). Available online at: https://www.adeccoinstitute.es/wp-content/uploads/2019/06/VIII-Informe-Absentismo.pdf (accessed Aug 21, 2020).

[25] Richard M-A Paul C De Pouvourville G Jullien D Mahe E Bachelez H. Out-of-pocket expenditures in France to manage psoriasis in adult patients: results from an observational, cross-sectional, non-comparative, multicentre study. J Eur Acad Dermatol Venereol. (2021) 35:912–8. 10.1111/jdv.17000 33073410

[26] España. Real Decreto 231/2020, de 4 de febrero, por el que se fija el salario mínimo interprofesional para 2020. BOE núm. 31, de 5 de febrero de 2020. (2020). Available online at: https://www.boe.es/eli/es/rd/2020/02/04/231 (accessed Feb 12, 2020).

[27] Ortega Eslava A. Guía de Evaluación Económica e Impacto Presupuestario en los Informes de Evaluación de Medicamentos. Madrid: SEFH, Sociedad Española de Farmacia Hospitalaria (2017).

[28] Instituto Nacional de Estadística. Gasto total, gastos medios y distribución del gasto de los hogares. Consumiciones en bares y cafeterías. (2020). Available online at: https://www.ine.es/dynt3/inebase/es/index.htm?padre=3777&capsel=3778 (accessed Jan 2, 2022).

[29] Instituto Nacional de Estadística. Encuesta Anual de Estructura Salarial 2016. Resultados Nacionales y por Comunidades Autónomas. Ganancia por hora normal de trabajo. (2016). Available online at: https://www.ine.es/jaxiT3/Datos.htm?t=28205 (accessed Dec 21, 2018).

[30] Strober B Papp KA Lebwohl M Reich K Paul C Blauvelt A. Clinical meaningfulness of complete skin clearance in psoriasis. J Am Acad Dermatol. (2016) 75:77–82.e7. 10.1016/j.jaad.2016.03.026 27206759

[31] Jankowiak B Kowalewska B Krajewska-Kułak E Khvorik DF. Stigmatization and quality of life in patients with psoriasis. Dermatol Ther. (2020) 10:285–96. 10.1007/s13555-020-00363-1 32146709 PMC7090112

[32] Schiffner R Schiffner-Rohe J Gerstenhauer M Hofstädter F Landthaler M Stolz W. Willingness to pay and time trade-off: sensitive to changes of quality of life in psoriasis patients? Br J Dermatol. (2003) 148:1153–60. 10.1046/j.1365-2133.2003.05156.x 12828743

[33] Seidler AM Bayoumi AM Goldstein MK Cruz PD Chen SC. Willingness to pay in dermatology: assessment of the burden of skin diseases. J Invest Dermatol. (2012) 132:1785–90. 10.1038/jid.2012.50 22418874

[34] Stefanidou M Evangelou G Kontodimopoulos N Koumaki D Krueger-Krasagakis S-E Yosipovitch G. Willingness to pay and quality of life in patients with pruritic skin disorders. Arch Dermatol Res. (2019) 311:221–30. 10.1007/s00403-019-01900-5 30788568

[35] Maymone MBC Rajanala S Widjajahakim R Secemsky E Saade D Vashi NA. Willingness-to-pay and time trade-off: the burden of disease in patients with benign hyperpigmentation. J Clin Aesthetic Dermatol. (2019) 12:46–8. 31320977 PMC6561712

[36] Graham CN Miles L McBride D Zhao Y Herrera V. Estimation of Annual Indirect Costs Associated With Moderate-to-Severe Plaque Psoriasis in the United States. (2016). 10.1016/j.jval.2015.09.547

[37] Lebwohl M Soliman AM Yang H Wang J Freimark J Puig L. Impact of PASI response on work productivity and the effect of risankizumab on indirect costs using machine learning in patients with moderate-to-Severe psoriasis. J Dermatol Treat. (2021) 33:1–26. 33899655 10.1080/09546634.2021.1919287

[38] Zozaya N Villoro R Abdalla F Alfonso Zamora S Balea Filgueiras J Carrascosa Carrillo JM. Unmet needs in the management of moderate-to-severe psoriasis in spain: a multidimensional evaluation. Acta Derm Venereol. (2022) 102:adv00678. 10.2340/actadv.v102.583 35312022 PMC9631248

[39] Wilder-Smith A Longini I Zuber PL Bärnighausen T Edmunds WJ Dean N. The public health value of vaccines beyond efficacy: methods, measures and outcomes. BMC Med. (2017) 15:138. 10.1186/s12916-017-0911-8 28743299 PMC5527440

[40] Luyten J Beutels P. The social value of vaccination programs: beyond cost-effectiveness. Health Aff Proj Hope. (2016) 35:212–8. 10.1377/hlthaff.2015.1088 26858372

[41] Sullivan J Shih TM van Eijndhoven E Jalundhwala YJ Lakdawalla DN Zadikoff C. The social value of improvement in activities of daily living among the advanced Parkinson's disease population. Forum Health Econ Policy. (2020) 23:1–23. 10.1515/fhep-2019-0021 33984886

